# Allicin promotes functional recovery in ischemic stroke via glutathione peroxidase-1 activation of Src-Akt-Erk

**DOI:** 10.1038/s41420-023-01633-5

**Published:** 2023-09-06

**Authors:** Fei Zhuang, Xin Shi, Sen Qiao, Bin Liu, Zhimei Wang, Huanhuan Huo, Feng Liang, Linghong Shen, Lijuan Zhu, Ben He, Hongmei Wang

**Affiliations:** 1 Shanghai Chest Hospital, School of Medicine, Shanghai Jiao Tong University, Shanghai, 200030 China; 2 Northwest Women’s and Children’s Hospital, Xi’an, 710003 China; 3 Graduate School, Bengbu Medical College, Anhui, 233000 China; 4 School of Medicine, Southeast University, Nanjing, 210009 China

**Keywords:** Molecular neuroscience, Astrocyte

## Abstract

Allicin exhibits various pharmacological activities and has been suggested to be beneficial in the treatment of stroke. However, the underlying mechanisms are largely unknown. Here, we confirmed that allicin protected the brain from cerebral injury, which could be ascribed to its anti‑apoptotic and anti‑inflammatory effects, as well as the regulation of lipid metabolism, using proteomics and metabolomics analysis. Our results suggested that allicin could significantly ameliorate behavioral characteristics, cerebral infarct area, cell apoptosis, inflammatory factors, and lipid metabolic-related factors (arachidonic acid, 15-hydroperoxy-eicosatetraenoic acid (15S-HPETE), palmitoylcarnitine, and acylcarnitine) by recalibrating astrocyte homeostasis in mice with photothrombotic stroke (PT). In astrocytes, allicin significantly increased glutathione peroxidase 1 (GPX1) levels and inhibited the arachidonic acid-related pathway, which was also observed in the brains of mice with PT. Allicin was proven to inhibit hypoxia-induced astrocyte apoptosis by increasing GPX1 expression, activating proto-oncogene tyrosine-protein kinase Src (Src)- protein kinase B (AKT)-extracellular signal-regulated kinase (ERK) phosphorylation, and decreasing lipid peroxidation. Thus, we concluded that allicin significantly prevented and ameliorated ischemic stroke by increasing GPX1 levels to complete the complex physiological process.

## Introduction

Stroke is one of the most vital vascular events in the central nervous system and is the second leading cause of death and disability in modern society [1]. The prevalence of stroke is highest in developing countries, and ischemic stroke is the most common type. Ischemic stroke is brain tissue infarction due to cerebral artery occlusion, accompanied by damage to neurons, astrocytes, and oligodendrocytes [2]. The conventional treatment for ischemic stroke is either thrombolytic therapy or surgical or mechanical removal of blood clots [2, 3]. However, these treatments have a limited time window, high recurrence rates, and limited neurological recovery. Therefore, there is an urgent need to develop preventative and effective treatments for ischemic stroke.

The activation of astrocytes is one of the important pathologic mechanisms of ischemic stroke [4]. In the early stage of ischemic stroke, activated astrocytes can maintain the homeostasis of the brain microenvironment by regulating energy metabolism and secreting neuroprotective factors. However, at later stages, activated astrocyte proliferation can inhibit the plasticity and regeneration of the central nervous system [5, 6]. The activation of astrocytes is regulated by a variety of factors. Previous studies have investigated inflammatory factors, transforming growth factors, neurotrophic factors, tumor suppressors, and other factors that can trigger the activation of astrocytes [7]. Recently, more attention has been paid to the effect of disordered lipid metabolism on astrocyte activation. For example, the dys-synthesis of cholesterol in astrocytes can lead to neuronal damage, while excess cholesterol can trigger the activation of astrocytes [8, 9]. The role of disordered lipid metabolism, especially that of astrocytes, in the pathological process of stroke remains unclear. Therefore, it is particularly important to study the influence of disordered lipid metabolism in ischemic stroke on astrocyte activation and to search for key regulatory molecules.

Allicin, with a broad range of biological activities, is a defensive molecule derived from garlic (*Allium sativum* L.). It is a reactive sulfur species (RSS), which causes a redox reaction with thiol groups in glutathione and proteins and is considered to be important for its biological activity [10, 11]. Allicin has been proven to inhibit the tumor growth of explants by regulating the Nrf2-system and ERK1/2 map kinases in immune cells of glioma [12, 13] and attenuate age-related cognitive and memory deficits by regulating the Nrf2-system [12, 14, 15]. Studies also demonstrated that allicin was beneficial to health because it could selectively reduce the levels of triacylglycerol, total cholesterol, and low-density lipoprotein (LDL)-cholesterol without any alteration in high-density lipoprotein (HDL)-cholesterol levels [16, 17]. Allicin was also shown to be effective in suppressing cholesterol biosynthesis, which is involved in ischemic diseases such as angina pectoris, myocardial infarction, and stroke [18, 19]. These reports suggest that allicin has a variety of health-promoting properties and may play vital roles in regulating lipid disorders. Understanding how allicin regulates their metabolism will have crucial therapeutic significance for ischemic stroke therapy and aid in developing new approaches to lipid metabolism.

Here, we examined the potential role of allicin in protecting the brain from cerebral injury, including ameliorating behavioral disorder, cerebral infarction areas, cell apoptosis, and regulating lipid metabolic-related factors in photothrombotic (PT) mice by brain omics assay. Since astrocytes play vital roles in stroke, how allicin modified astrocytes and the mechanisms by which they regulated ischemic stroke were also studied in a context-dependent manner. We also proposed an alternative explanation for the pathogenic role of allicin in ischemic stroke by promoting protective genes and included a detailed potential discussion.

## Results

### Allicin improves post-stroke functional impairment, including behavior and cerebral infarct size

**Fig. 1:**
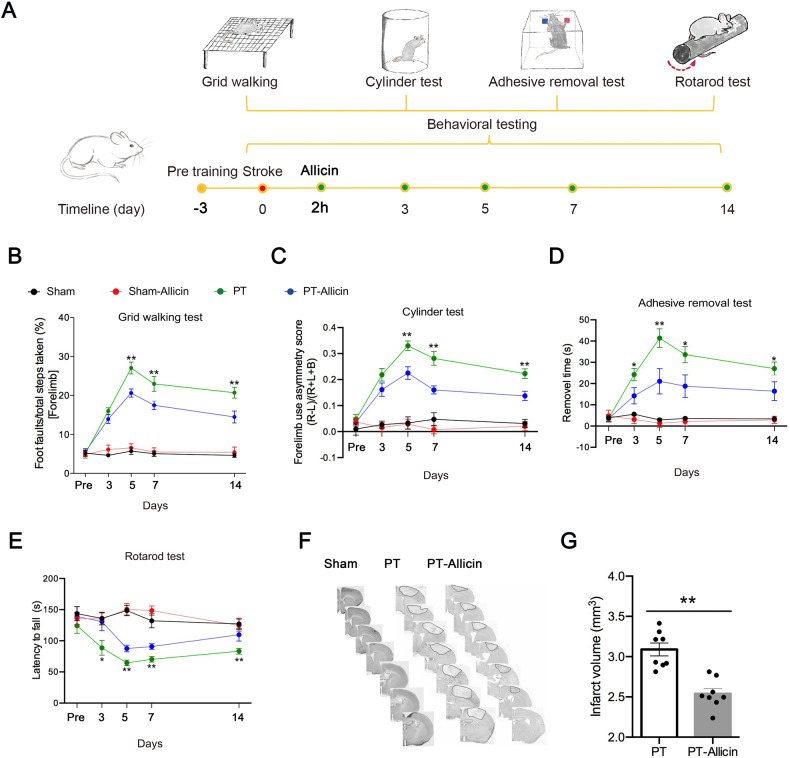
Allicin inhibited the behavioral damage of ischemic stroke in PT mice. **A** Schematic of allicin administration and behavioral tests at different time points. **B**–**E** Allicin improved the recovery of the PT mice behavior disorder as measured by the test of **B** grid-walking, **C** cylinder, **D** adhesive removal, and **E** rotarod. *n* = 14 animals/group. **F**, **G** The representative Nissl stained sections were obtained from PT or PT + Allicin mice, and allicin obviously reduced the cerebral infarction area of PT mice. *n* = 8 animals/group. *P < 0.05, **P < 0.01, PT versus the PT + Allicin.

Previous studies showed that allicin could reduce cerebral infarction areas, neuronal apoptosis, myeloperoxidase (MPO) activity, and tumor necrosis factor (TNF)‑α levels in serum to protect the brain from cerebral ischemia/reperfusion injury [7]. To further understand the potential roles of allicin in stroke, allicin was administered to mice by intraperitoneal injection 2 h after PT stroke daily for 14 consecutive days, and behavioral testing was performed on days 3, 5, 7, and 14 after stroke (Fig. 1A). Animals in the PT-allicin group showed improved behavior and decreased foot faults on days 3, 5, 7, and 14 in a grid-walking test compared to the PT group (Fig. 1B). The cylinder test revealed similar results, and the mice injected with allicin showed improved performance on days 3, 5, 7, and 14 after PT stroke (Fig. 1C). The same therapeutic effect was also found in the adhesive removal test (Fig. 1D) and the rotarod test (Fig. 1E). Therefore, the results suggested that treatment with allicin promoted behavioral recovery. Nissl staining was assessed on day 5, and the results showed an obvious decrease in the infarct size between the PT group and the PT-allicin group (Fig. 1F, G).

### Proteomic analysis of differentially expressed proteins in the allicin-treated peri-infarct cortex

**Fig. 2:**
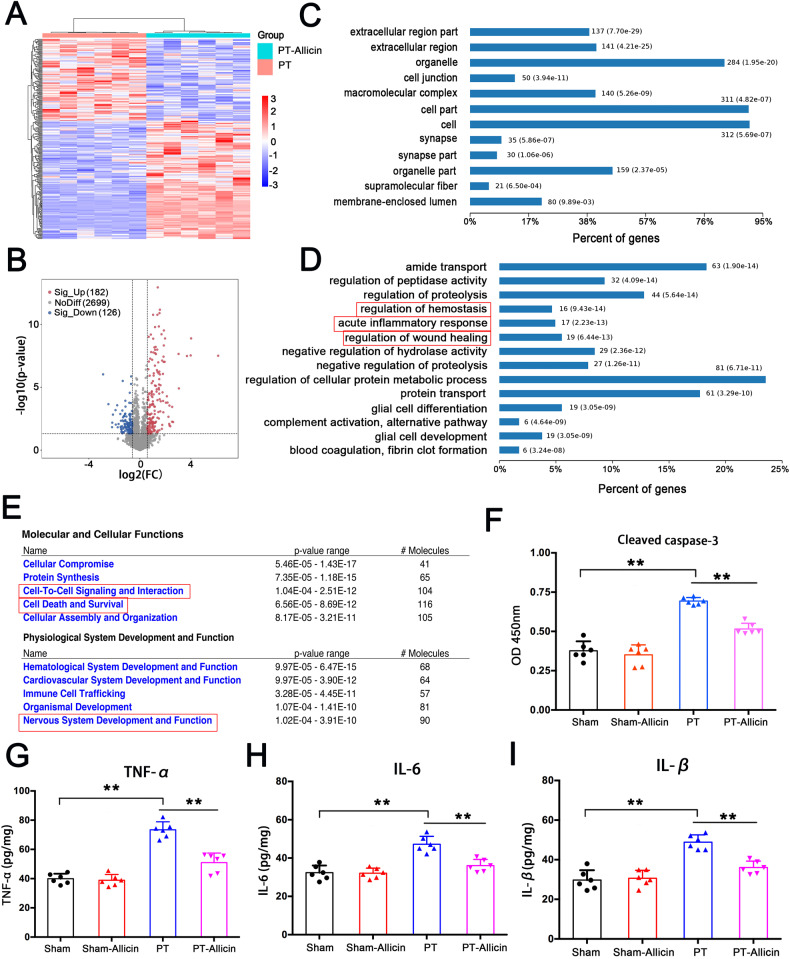
Quantitative proteomic analysis of the differential expressed proteins between PT and PT + Allicin groups. **A** Hierarchical clustering analysis revealed 208 differential expressed proteins (at least FC > 2 and *p* < 0.05) were detected in three repeats of two groups (PT and PT + allicin). **B** The volcano chart showed 2699 protein showed no significant difference (gray dots). Meanwhile, compared with the PT group, 162 proteins (red dots) were upregulated, and 128 proteins (blue dots) were downregulated in PT + Allicin group. **C**, **D** Cell component and biological process of GO enrichment analysis and (**E**) IPA analysis for the categories of differently regulated proteins between PT + allicin and PT groups. **F** The expression level of cleaved caspase-3 in the surrounding area of cerebral infarction of PT mice with allicin treatment analyzed by Elisa Kit. **G**–**I** Allicin inhibited the expression of inflammatory factors (TNFα, IL-6, and IL-1β) in PT + Allicin mice. *n* = 6 animals/group.

The protein composition of the peri-infarct brain area was analyzed by liquid chromatography with liquid chromatography-tandem mass spectrometry (LC–MS/MS)-based shotgun proteomic approach combined with bioinformatics analysis to reveal the therapeutic effect of allicin on post-stroke. Proteomic analysis identified 3007 proteins in both the PT and PT-allicin groups and the heatmap (Fig. 2A) and principal component analysis (PCA) analysis (Supplementary Fig. 1A) demonstrated that the protein composition of the peri-infarct brain area was significantly changed by allicin. Volcano plots further revealed 308 proteins with significant differences between the two groups (*P*-value < 0.05 and fold-change > 2). Of these, 182 proteins were upregulated and 126 proteins were downregulated (Fig. 2B, Supplementary Table S1). Therefore, these results suggested that allicin changed the protein component of the peri-infarct cortex.

Bioinformatics analysis was then applied to investigate the potential biological functions of the differentially expressed proteins and the possible mechanisms involved in the therapeutic effects of allicin on the post-stroke condition. Gene Ontology (GO) was used to analyze the cell component (CC) and biological processes (BP) in which these 308 differentially expressed proteins may be involved. CC analysis demonstrated that the differential proteins were mainly found in the extracellular region, cell junctions, and synapses, which indicated the reliability of the sample source (Fig. 2C). The BP analysis results revealed that the differentially expressed proteins were significantly enriched in the regulation of hemostasis, acute inflammatory response, and the regulation of wound healing, suggesting that allicin may regulate the homeostasis of the infarcted area and participate in the inflammatory response and repair of the infarcted area (Fig. 2D).

Ingenuity pathways analysis (IPA) was applied to demonstrate the molecular and cellular function of the differentially expressed proteins. The results suggested that the differentially expressed proteins might be involved in cell-to-cell signaling and interaction, as well as cell death and survival. The developmental and functional analysis of the physiological system showed that the differentially expressed proteins affected nervous system development and function (Fig. 2E). The Gene Set Enrichment Analysis (GSEA) analysis results suggested that anti-inflammatory and antioxidant phosphorylation pathways played an important role in allicin regulation (Supplementary Fig. 1B, C). Overall, the proteomic results suggested that allicin might improve cerebral infarction in mice by regulating the nervous system, cell death and survival, inflammatory response, and oxidative phosphorylation.

Cleaved caspase-3 was analyzed to confirm the role of allicin in cell death and survival and to confirm the proteomic analysis results (Fig. 2F). The results showed that allicin significantly reduced the expression of cleaved caspase-3 in mice with cerebral infarction, but no significant effect was observed in the sham operation group. We also analyzed the inflammation-related factors TNF-α, interleukin (IL)-6, and IL-1β, which were obviously attenuated by allicin, indicating that allicin could inhibit the inflammatory response in the cerebral infarct area (Fig. 2G–I). These results confirmed the beneficial effects of allicin on cell death and survival and the inflammatory response.

### Allicin changes arachidonic acid-related lipid metabolism in the peri-infarct cortex

**Fig. 3:**
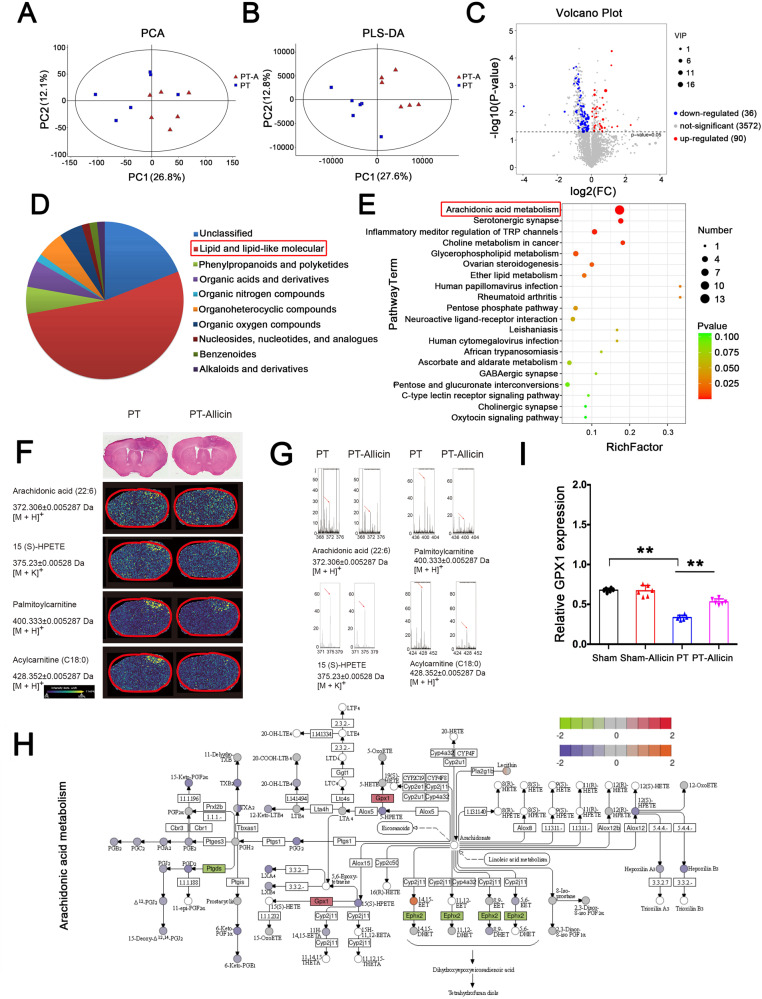
Quantitative metabonomics analysis reveals distinct metabolites between PT and PT+Allicin groups. **A**, **B** Surrounding area of cerebral infarction from PT mice after molding was used for metabonomics analysis. PCA and PLS-DA plot analysis showed the distinct metabolite between PT and PT + Allicin groups. **C** Volcano plots revealed that 3572 metabolites showed no significant difference and 126 metabolites showed significant differences, with 90 upregulated and 36 downregulated VIP (variable important in projection) > 1.0 and *p*-value < 0.05). **D** The percentile pie chart showed the proportion of different types of metabolites. Among these, lip and lip-like molecules were the most abundant. **E** KEGG analysis of the differential metabolite (TOP20) between PT and PT + Allicin groups. **F** Spatial distribution of identified masses in ion modes on brain sections of PT and PT + Allicin groups, and the representative imagines of matched H&E staining in brains (Magnification × 4, Scale bars, 100 μm). **G** Classic mass spectrometry in brain samples. **H** Combined proteomic and metabolomic analysis revealed that GPX1 affected arachidacida-related metabolic pathways. **I** The expression level of GPX1 in the surrounding area of cerebral infarction of PT mice with allicin treatment was analyzed by Elisa Kit, and the results revealed that allicin obviously increased GPX1 expression.

A broad-spectrum metabolomics analysis of the cerebral infarction area of mice was conducted to investigate the effect of allicin on cerebral infarct metabolomics. PCA and partial least squares discriminant analysis (PLS-DA) were performed. The results showed that the metabolite expressions were different between the PT and PT-allicin groups (Fig. 3A, B). The volcano plot of the metabolomic analysis further revealed that 36 metabolites were significantly upregulated, and 90 metabolites were downregulated (Fig. 3C, *p* < 0.05, FC > 2, Supplementary Table S2). Next, we classified these 126 differentially expressed metabolites and found that the highest one was lipid and lipid-like molecules (Fig. 3D). Kyoto Encyclopedia of Genes and Genomes (KEGG)-based analysis showed that the differentially expressed metabolites were involved in arachidonic acid metabolism, the glycerophospholipid pathway, and inflammatory mediator regulation of Transient receptor potential channel (TRP) channels, suggesting that allicin may change the metabolism in the peri-infarct cortex, especially arachidonic acid metabolism (Fig. 3E). Therefore, the role of arachidonic acid metabolism in the peri-infarct cortex was further studied.

Recent studies demonstrated that alterations in lipid metabolism, especially arachidonic acid metabolism like PGE2, 5-HPETE, TXA2, and TXB2, influenced the inflammatory response in post-stroke mice, and MALDI-imaging was then used to identify the expression of lipid peaks in the brain (Supplementary Fig. 2). The expression intensities of SM peaks with *m*/*z* values of 372.301, showing that arachidonic acid was slightly reduced in the peri-infarct cortex of the PT-allicin group compared to the PT group. A series of metabolites related to arachidonic acid metabolisms, such as paimitoylcarnitine with an m/z value of 400.312, acylcaritine with 428.352 *m*/*z* values of 372.301, and 5-HPETE with an *m*/*z* value of 375.230, were significantly reduced in the PT-allicin group (Fig. 3F, G). The MALDI-imaging results were consistent with those of the MALDI–TOF–MS analysis and suggested that allicin altered lipid metabolism in the cerebral infarct area, especially arachidonic acid metabolism, which may be involved in post-stroke functional impairment.

We performed combined metabolomics and proteomics analysis between the PT-allicin and PT groups to further explore the underlying molecular mechanisms of arachidonic acid-related lipid metabolism in stroke models. GPX1 was shown to regulate arachidonic acid-related lipid metabolism and was up-regulated by allicin in the PT-allicin group. Confirmatory GPX1 enzyme-linked immunosorbent assay (ELISA) Kit analysis was then performed, and the results demonstrated that GPX1 expression in the PT group was significantly decreased compared to the sham group, whereas allicin significantly increased the expression level of GPX1 in the infarct area of mice with cerebral infarction, consistent with our proteomic results (Fig. 3H, I). These findings suggest that GPX1 in the peri-infarct zone may play a vital role in stroke recovery.

### Knockdown of GPX1 aggravates motor coordination and increases the size of cerebral infarction in post-stroke mice

**Fig. 4:**
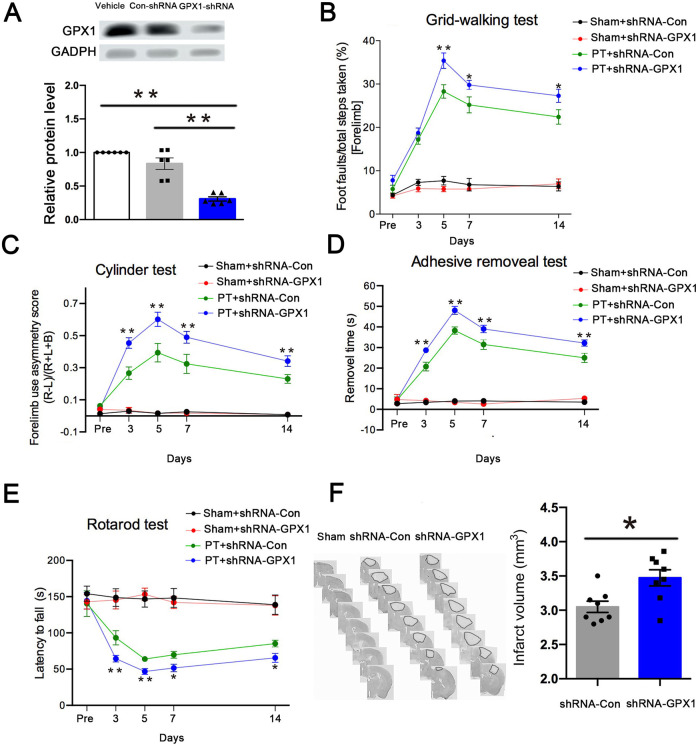
Knockdown of GPX1 aggravated motor coordination and increased the size of cerebral infarction in post-stroke mice. **A** Western blot showed the deletion of GPX1 in the cerebral cortex of mice. *n* = 6 animals/group. **B**–**E** Stroke (PT) or sham surgery (Sham) mice injected with shRNA-GPX1 or shRNA-Con were subjected to behavioral tests. Repeated measure task **B** grid-walking test, **C** cylinder test, **D** adhesive removal test, and **E** rotarod test were significantly different between the sham-shRNA-GPX1 and PT-shRNA-GPX1 groups. Knockdown GPX1 significantly deteriorated motor coordination in the mice after stroke. *n* = 10 animals/group. **F** Knockdown GPX1 increased the size of cerebral infarction after stroke. Representative images showed Nissl’s staining extravasation in the brain parenchyma. shRNA-GPX1group showed a significant expansion in cerebral cortex infarct volume in brain parenchyma compared with the vehicle-treated group. *n* = 8 animals/group. *p < 0.05, **p < 0.01; PT + shRNA-GPX1 vs. PT + shRNA-Con.

The lentiviral vector shRNA-GPX1 and its control shRNA-Con were used to further demonstrate the effect of GPX1 on stroke recovery. We injected shRNA-GPX1 or shRNA-Con into the prospective stroke site of mice. After three days, Western blot analysis was used to assess the efficacy of lentivirus transduction (Fig. 4A). Behavioral testing was conducted pre-stroke and on days 3, 5, 7, and 14 after the stroke, and the volume of the brain infarct was measured. The present study indicated that ischemia aggravated behavioral function, including the grid-walking test, cylinder test, adhesive removal test, and rotarod test, in the PT group compared to the sham group. Moreover, treatment of PT mice with shRNA-GPX1 aggravated ischemia-impaired behavior (Fig. 4B–E). In addition, the knockdown of GPX1 in PT mice caused significant increases in the volume of the brain infarct after stroke (Fig. 4F). Therefore, GPX1 plays a vital role in post-stroke functional impairment, and allicin may improve this impairment by increasing GPX1 expression.

### Allicin inhibits astrocyte apoptosis by increasing GPX1 expression, activating Src-Akt-Erk phosphorylation

**Fig. 5:**
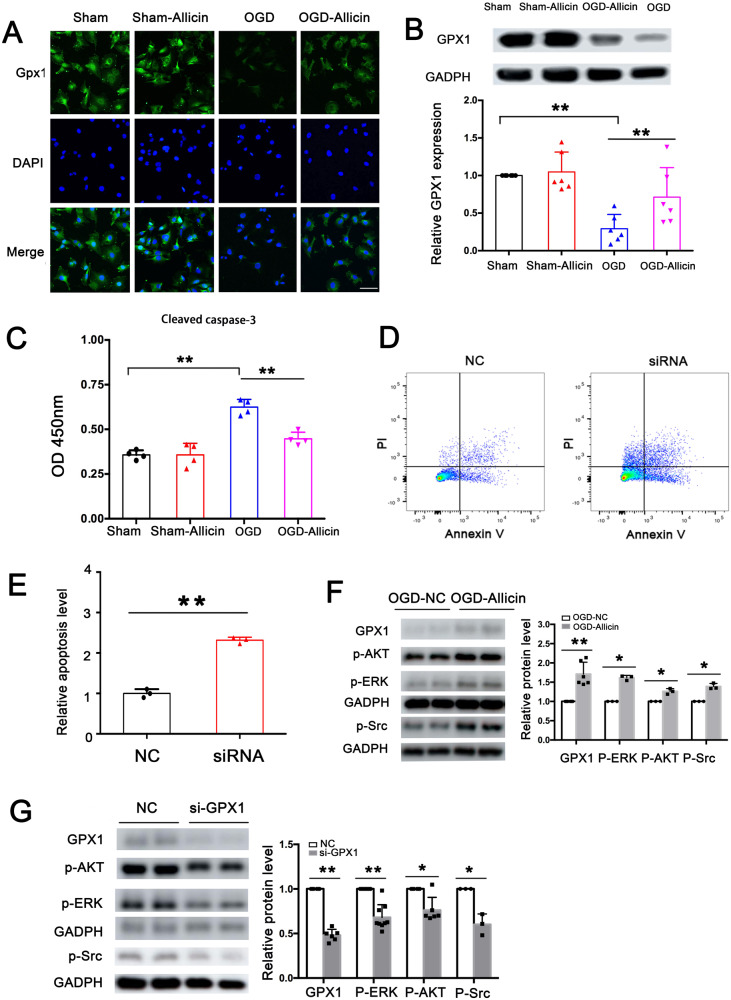
Allicin inhibited astrocyte apoptosis by increasing GPX1 expression, activating Src-Akt-Erk phosphorylation. **A** Immunofluorescence assay showed that allicin could reverse OGD-induced GPX1 decrement but had no significant effects on the sham group. Bar = 20 μm. **B** Western blots further confirmed that allicin effectively reversed the inhibitory effect of hypoxia on GPX1 expression. *n* = 6. **C** Cleaved caspase-3 Elisa kit was used to detect the apoptosis of the astrocytes, and the results revealed that allicin significantly decreased cleaved caspase-3 expression. *n* = 4. **D**, **E** siRNA-GPX1 was used to interfere with GPX1 expression, and the Annexin V/PI kit was then used to detect the apoptosis of astrocytes. Flow cytometry results showed that siRNA-GPX1 obviously increased astrocyte apoptosis. *n* = 3. **F** Western blotting analysis showed that allicin promoted GPX1 expression in the OGD-Allicin group and obviously increased Src-Akt-Erk phosphorylation compared with the OGD-NC group. *n* = 6. **G** Western blotting analysis revealed that siRNA-GPX1 obviously decreased Src-Akt-Erk phosphorylation compared with the NC group. *n* = 6.

Since astrocytes played a vital role in the inflammatory and antioxidant responses, the response of astrocytes to allicin treatment was explored. Immunofluorescence experiments suggested that GPX1 expression was significantly inhibited by hypoxia treatment, whereas allicin increased GPX1 expression under hypoxic conditions but had no significant effect on normal cells (Fig. 5A), consistent with animal experiment results. Western blot results further confirmed that allicin effectively reversed the inhibitory effect of hypoxia on GPX1 expression (Fig. 5B).

The apoptosis of astrocytes was detected with the cleaved caspase-3 ELISA kit to explore whether allicin could regulate astrocyte apoptosis. The results showed that hypoxia increased astrocyte apoptosis, whereas allicin reversed hypoxia-induced astrocyte apoptosis (Fig. 5C). As allicin increased GPX1 expression under hypoxic conditions and GPX1 played a vital anti-apoptotic role, we speculated that allicin could inhibit astrocyte apoptosis by increasing GPX1 expression in astrocytes under hypoxic conditions. To further confirm this hypothesis, siRNA was used to interfere with the expression of GPX1 in astrocytes under hypoxic conditions. The annexin V/propidium iodide (PI) kit was then used to detect the apoptosis of astrocytes. The results revealed that astrocyte apoptosis was significantly increased in the GPX1-siRNA group (Fig. 5D, E). Thus, the present results suggested that allicin may inhibit astrocyte apoptosis by increasing GPX1 expression.

Since the Src-Akt-Erk axis was shown to play a vital role in decreasing astrocyte apoptosis, we hypothesized that allicin-stimulated GPX1 up-expression activated the Src-AKT-ERK axis and played an anti-apoptotic role in astrocytes after OGD treatment (Fig. 5F). Confirmatory Western blotting analysis showed that the allicin-induced expression of GPX1 in the OGD-allicin group significantly activated Src-Akt-Erk phosphorylation compared to the OGD-NC group. Treatment with siRNA-GPX1 after allicin treatment significantly inhibited Src-Akt-Erk phosphorylation compared to the NC group (Fig. 5G). Thus, allicin could inhibit astrocyte apoptosis by increasing GPX1 expression and activating Src-Akt-Erk phosphorylation.

### Allicin inhibits hypoxia-induced astrocyte lipid metabolism peroxidation

**Fig. 6:**
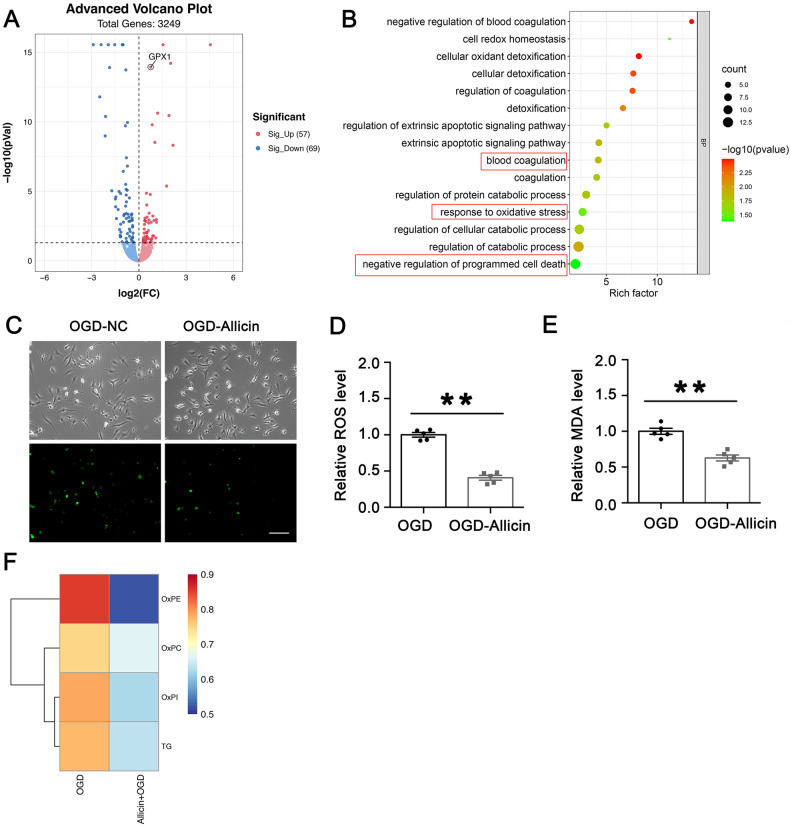
Allicin inhibited hypoxia-induced astrocyte lipid metabolism peroxidation. **A** Volcano plots of OGD+Allicin/OGD protein comparisons in astrocytes. Compared with the OGD group, 57 proteins (red clots) were obviously upregulated, including the focused protein GPX1, and 69 proteins (blue clots) were significantly downregulated in the OGD-Allicin group. **B** BP analysis for the categories of differently expressed proteins between OGD + Allicin and OGD groups. **C**, **D** Allicin obviously inhibited the intracellular ROS levels of astrocytes in the OGD-Allicin group. *n* = 5. **E** MDA assay results revealed that the MDA level of astrocytes in the OGD-Allicin group was obviously decreased in comparison with the OGD group. *n* = 5. **F** lipid metabolomics assay was carried out, and the results showed the average expression level of ox-phosphatidylethanolamines (ox-PE), ox-phosphatidylcholines (ox-PC), ox-phosphatidylinositols (ox-PI) and triacylglycerols (TG) level decreased in OGD-Allicin group. *n* = 6.

Allicin-stimulated astrocytes treated with OGD were collected for MS analysis to further explore the role of allicin in astrocytes. A total of 3249 proteins were identified from the two different groups. The volcano plots identified 126 differentially expressed proteins between the OGD and OGD-allicin groups (Fig. 6A, *p* < 0.05; Supplementary Table S3). There were 57 upregulated proteins in allicin-treated astrocytes, including GPX1, and 69 downregulated proteins compared to the control group. BP analysis was performed to functionally analyze the proteins differentially expressed in astrocytes between the OGD and OGD-allicin groups. The results showed that the differentially expressed proteins were mainly involved in the oxidative stress response, blood coagulation, and negatively regulated programmed cell death (Fig. 6B).

The intracellular reactive oxygen species (ROS) levels of astrocytes pretreated with allicin were exposed to OGD to explore the effect of allicin on the oxidative stress response. The results showed that allicin inhibited the intracellular ROS levels of astrocytes (Fig. 6C, D). The MDA assay results showed that MDA levels were decreased in the OGD-allicin group compared to the OGD group (Fig. 6E). The lipid metabolomics assay revealed that ox-phosphatidylethanolamines (ox-PE), ox-phosphatidylcholines (ox-PC), ox-phosphatidylinositols (ox-PI), and triacylglycerols (TG) levels were also decreased in the OGD-allicin group (Fig. 6F), suggesting that allicin decreased astrocyte lipid peroxidation.

### Allicin improves post-stroke functional impairment, including behavior and cerebral infarction area, by increasing GPX1 levels

**Fig. 7:**
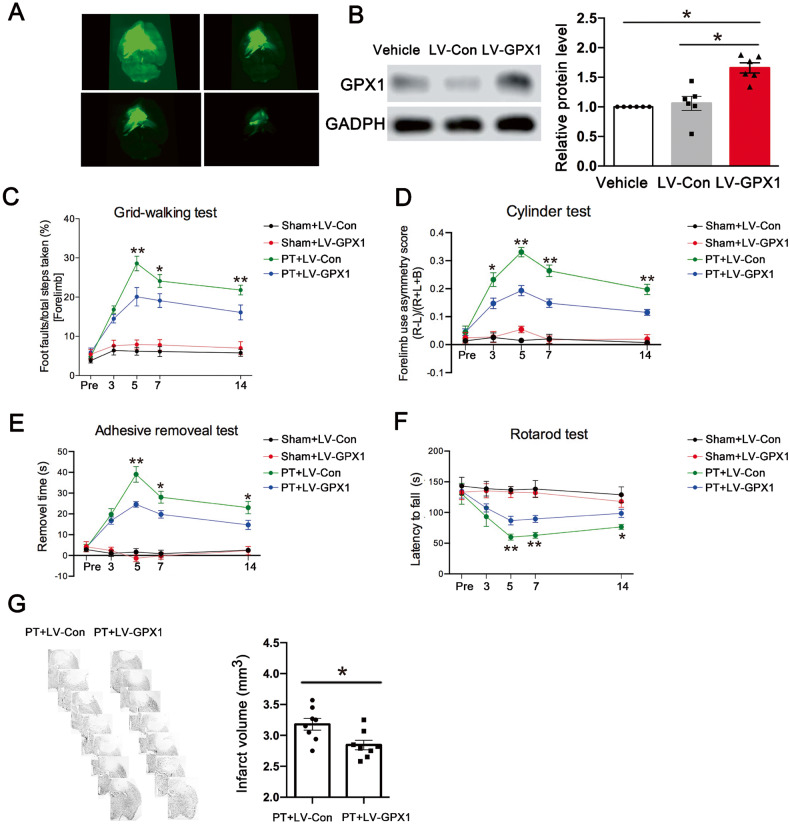
Overexpression GPX1 ameliorated motor coordination and increased the size of cerebral infarction in post-stroke mice. **A** 3D stereofluorescence determination of GPX1 overexpression after stereoscopic localization of lateral ventricles. **B** Western blot showed the expression level of GPX1 in the cerebral cortex of mice. *n* = 6 animals/group. Repeated measure task **C** grid-walking test, **D** cylinder test, **E** adhesive removal test, and **F** rotarod test ameliorated motor coordination in PT + LV-GPX1 group. Overexpression GPX1 significantly improved motor coordination in the mice after stroke. *n* = 10 animals/group. **G** Overexpression GPX1 reduced the size of cerebral infarction after stroke. *n* = 8 animals/group. Data are mean ± SEM. *p < 0.05, **p < 0.01; PT + LV-GPX1 vs. PT + LV-Con.

Previous studies showed that allicin may protect the brain from cerebral ischemia/reperfusion injury. Mice in the PT group with allicin stimulation were used to explore the potential mechanism of action of GPX1 in stroke. An LV-GPX1-overexpressing virus was constructed and verified by injecting it into the brains of normal mice. The brain scan showed that LV-GPX1 was effectively expressed (Fig. 7A). After that, LV-GPX1 was injected into the PT mice, and the Western blotting results showed that LV-GPX1 also effectively infected the cerebral infarction (Fig. 7B). The animal behavior test was then performed in mice administered LV-GPX1. They showed improved performance on the cylinder test on days 3, 5, 7, and 14 after PT surgery compared to the LV-Con group (Fig. 7C–F). We also found therapeutic effects of LV-GPX1 in the grid-walking test, adhesive test, and rotarod test after PT surgery. LV-GPX1 also significantly decreased the volume of the brain infarct after stroke (Fig. 7G). Hence, the results suggested that LV-GPX1 treatment promoted motor recovery in the PT group.

## Discussion

This study explored the role of allicin in stroke and demonstrated that the administration of allicin improved behavior recovery after stroke and enhanced neuroplasticity by regulating GPX1. Investigations into the possible mechanism showed that allicin inhibited the apoptosis of astrocytes induced by hypoxia via GPX1, activating SRC-AKT-ERK and lipid peroxidation. Accumulating evidence demonstrated that allicin had therapeutic effects in PT mice.

The development of therapeutic drugs for stroke has been limited due to complex challenges. The presence of the blood–brain barrier (BBB) is one of the major obstacles and strictly inhibits the delivery of therapeutic drugs into the brain. Conventional approaches using free-form drug molecules have shown poor BBB penetration. One of the main reasons was that efflux pumps on endothelial cells strictly regulate the movement of drug molecules [20, 21]. Hence, drug delivery is particularly important in current stroke treatment. TRPV channels were reported to be activated by naturally occurring pungent molecules, such as allicin, capsaicin, and resiniferatoxin [22, 23]. We also found that allicin activated the ion channel TRPV2 in astrocytes using the patch–clamp technique (Supplemental Fig. 3). Allicin increased the frequency of discharge while the TRPV2 channel blocker tranilast (75 μm) inhibited the frequency of discharge traces, which were induced by allicin. Based on our research, allicin, when used therapeutically, may be delivered into the brain by TRPV2 to improve post-stroke functional impairment, including behavior and the cerebral infarction area.

Allicin has the ability to resist cancer, microbial, and oxidant properties and is also used as an efficient therapeutic drug for cardiovascular disease treatment [24, 25]. In this context, our study revealed that allicin was a neuroprotective and antioxidant molecule that could improve behavior recovery in neurodegenerative disorders, such as stroke. BP analysis revealed that allicin was involved in oxidative stress, blood coagulation, and the negative regulation of programmed cell death (Fig. 6B). Moreover, allicin inhibited the apoptosis of astrocytes by activating SRC-AKT-ERK via GPX1-dependent pathways (Fig. 4). Allicin protected against astrocyte damage in ischemic stroke by inhibiting inflammation and apoptosis and increasing the expression levels of GPX1.

Lipid metabolism balance is an important regulatory factor for the steady state of the nervous system. Clinical studies showed that abnormal lipid metabolism was one of the main causes of brain degeneration after ischemic stroke [26]. Our study revealed that allicin altered lipid metabolism in the peri-infarct cortex and decreased arachidonic acid metabolism like PGE2, 5-HPETE, TXA2, and TXB2 (Fig. 3), which inhibited inflammatory responses in post-stroke mice. Moreover, allicin decreased total cholesterol (TC) levels in the allicin-treated peri-infarct cortex (Supplementary Fig. 4). Since a large amount of cholesterol enters vascular endothelial cells and promotes the formation of foam cells and the development of vascular lesions, which leads to the occurrence and development of stroke [27, 28], allicin-induced decreases in TC levels might exert protective effects in ischemic stroke.

Although the present results indicated that allicin improved behavioral damage caused by ischemic stroke in PT mice, it should be noted that the sample size was small, which is susceptible to false positive results. Also, despite the use of randomized trial designs and blind assessors for sample groupings, differences between multi- and monocentric mice often encountered in preclinical randomized trials must be recognized. Based on our research, the present study supported that allicin has great potential for clinical application after stroke and could be regarded as a promising therapeutic strategy for behavior recovery.

## Materials and methods

### Ethical approval

C57 mice were generated at the National Resource Center of Mutant Mice/Model Animal Research Center of Nanjing University, Nanjing, Jiangsu, China. Animals were housed in cages with access to food and water under a standard 12/12 h light/dark cycle. All the experimental procedures were performed according to the approval of the animal welfare committees of Southeast University, Nanjing, Jiangsu, China. We also made efforts to reduce the number of animals used in the experiment and minimize animal suffering.

### Photothrombotic stroke

Light was used to induce thrombi in mice cortical microvessels, causing a localized stroke coma, as described previously. Briefly, mice anesthetized with 2% isofluoroalkanes were put into a stereotaxic frame (Wood Dale, USA). The skull was exposed through a midline incision in the skin at the top of the brain. A cold light source with a 2-mm-diameter fiber optic bundle (12,000 lux; World Precision Instruments, USA) was centered 1.5 mm lateral from the bregma. The brain was irradiated for 5 min after the mice were injected intravenously with Bengal Rose (30 mg/kg, Sigma Aldrich, USA). By light irradiation, Bengal Rose produced singlet oxygen, damaged and occluded the vascular endothelium, causing local ventricular cortex damage [29]. We then returned the animals to the cage and monitored them after regaining consciousness. In the non-irradiated sham group, we injected the same amount of Rose Bengal without any illumination. The mice were randomly assigned for follow-up experiments.

### Experimental grouping design

Pretrained male mice were randomly allocated into four groups (sham, sham-allicin, PT, and PT-allicin). Mice in the sham-allicin and PT-allicin groups were injected with 50 mg/kg of allicin 2 h after surgery and once daily for 14 days. Mice in the sham and PT groups were injected with the same volume of normal saline daily. Behavioral testing was then assessed on days 3, 5, 7, and 14 after PT stroke. After that, the mice were deprived of food overnight and anesthetized. The brain was dissected and frozen immediately in liquid nitrogen for further experiments.

### Grid-walking task

A 32 cm × 20 cm elevated grid manufactured with 1.2 cm square wire mesh was used for the grid-walking task. A camera was placed under the mesh to record defects in the animal’s foot. Each mouse was put on the surface of the wire and walked freely for 4 min. The foot fault and non-fault numbers per left forelimb were calculated. If the left forelimb of the mice dropped through the hole of the grid, it was defined as a foot fault. It should also be noted that if the wrist of the mouse stayed at the mesh level, it was also thought to be a foot fault. The total steps included the foot fault number and the non-fault number. The ratio of foot faults to total steps for the left forelimbs of the mice was then calculated” or “The ratio of foot faults to total left forelimb steps was then calculated [30].

### Cylinder task

The cylindrical task encouraged the mice to explore the vertical walls of the cylinder using their forelimbs, as previously described [31]. When the mice were placed into the plastic cylinder (height 15 cm, diameter 10 cm), they spontaneously pressed the cylindrical wall with one or two of their forelimbs to rear up. Each mouse was held in the cylinder for 5 min and recorded. We then calculated the number of times each mouse used the left forelimb, the right forelimb, or both forelimbs by parsing the video segments. The asymmetrical index was calculated as follows: right forelimb usage rate and left forelimb usage rate.

### Adhesive removal test

The adhesive removal test of the mice was assessed based on a previously described method [32]. Two small pieces (25 mm^2^) of adhesive-backed paper dots were used in this experiment to stimulate the bilateral tactile sensing of the mice and set on the distal-radial region of each forearm wrist. The time that the mice spent removing each small piece from the forelimb was recorded. The mice were trained for three days to be able to remove the spot within 10 s. The results were expressed as left forelimb time and right forelimb time.

### Rotarod test

A rotary machine (MK-660D, Muromachi-Kikai, Japan) with an automatic timer and falling sensor was used. First, we put the mouse on a drum 3 cm in diameter. Before training, the mouse had stayed on the stationary drum for 3 min. The practice was repeated for 1 min every day before starting training. Next, we set the rotation to a relatively slow speed (10 RPM/min) to make it easier for animals to complete the tasks. As soon as the animal fell, it was put back on the drum at once, up to five times in one session. Once the animal stayed on the drum for 180 s, a fall was ignored. The test was repeated once a day for four consecutive days to evaluate long-term memory. Finally, the behavioral test was performed [33].

### Infarct volume measurement

A sliding freezing stage microtome (Leica, Germany) was used to cut the mice brains into 50-μm coronal slices on day 5 after stroke. The infarct volume of the mice was stained with Nissl stain according to the manufacturer’s instructions (Beyotime, China), and a histological assessment was conducted. The infarct volume was assessed by an investigator blinded to group allocation using ImageJ. Measurements were taken of every sixth section throughout the entire infarct as follows: infarct volume (mm^3^) = infarct area (mm^2^) × slice thickness × slice interval [34].

### Comparative proteomic analysis

Brain tissue in a 3-mm area surrounding the cerebral was collected into a tube. After washing with PBS, the brain tissue was ground using a pestle under liquid nitrogen conditions. The treated cells were lysed on ice in cold-modified RIPA buffer and incubated for 30 min. LC/MS and protein analysis were then performed according to standard methods [35, 36]. GSEA was used to analyze the pathways in which the total detected proteins were involved. The cellular components and biological processes of the differentially expressed proteins requiring at least two peptides per protein were then analyzed by GO enrichment, and the candidate-related pathways were disclosed by KEGG analysis. Molecular and cellular function, as well as physiological system development and function, were analyzed using IPA software (content version: 65367011, http://www.ingenuity.com/products/ipa) [37].

### Cleaved caspase-3 activity assay

A cleaved caspase-3 ELISA kit (Cell Signaling Technologies, USA) was used to measure endogenous levels of cleaved caspase-3 protein in vivo and in vitro. Briefly, astrocytes or brain tissue lysis buffer were centrifuged at 14,000 rpm for 10 min, and the astrocyte supernatant was transferred to a new tube and analyzed according to the manufacturer’s instructions. Absorbance was assessed at 450 nm within 30 min using an ELISA plate reader. The results were described as activity relative to that in the control group.

### IL-6, IL-1β, and TNF-α assay

The expression of IL-6, IL-1β, and TNF-α in cells or tissue lysates was assessed using commercially available ELISA kits (R&D Systems, UK) according to the manufacturer’s instructions. The results were corrected by the protein concentration of the lysate determined using the BCA Protein Assay Kit (Beyotime, China) and described as activity relative to that of the control group.

### Sample preparation for MALDI-MS

Mouse brain samples are obtained from 8 to 10-week-old C57 male mice. After euthanizing, the brain was rapidly extracted and directly frozen in liquid nitrogen for further use. Brain sections were transferred by thaw-mounting on common microscopy glass slides (Superfrost, Thermo Fisher Scientific, Germany) for later LMD (P.A.L.M. Microlaser Technologies, Germany) [38]. All brain slices in this study continuously cut from the same brain of the mouse were used for H&E staining and the MALDI–MS experiment. 2.5-Dihydroxybenzoic acid (DHB) was used as the MALDI matrix. A total of 20 mg/ml of DHB dissolved in acetonitrile/water/methanol mixed with 0.1% formic acid was used for the subsequent spray-coating of the sample. The detailed protocol followed the O’Rourke MB manual [10].

### Metabolite extraction of the area surrounding cerebral infarction in mice

Tissue metabolites were extracted with some modifications of a previously described method [39]. After adding 800 μL of cold MeOH/H2O to the frozen tissue, a pellet pestle (Sigma-Aldrich, UK) was used to homogenize the tissue for 1 min. The samples were then centrifuged at 7000*g* for 5 min, and the supernatant was collected into a new tube for further use. Cold methanol/water (400 μL, −20 °C, 80:20 (v/v)) was added to the pellet, and the pellet was re-extracted. The supernatant was dried using a gentle flow of N_2_ gas and stored at −80 °C until dissolution for NMR analysis.

### Multivariate analysis of tissue extracts

PCA was conducted on samples to determine anomalies in the data and differences between the groups. To maximize separation between the classes, the NMR data were monitored by the dimension-reduction technique, i.e., the maximum margin criterion, using leave-one-out cross-validation with the quadratic to check the validation of the MMC model. One-way ANOVA analysis with a false discovery rate (FDR) of 0.1, i.e., 10%, was applied to determine the metabolites responsible for the class separation.

### Oxygen and glucose deprivation

The SVGp12 normal human astrocyte cell line (American Type Culture Collection, ATCC, USA) was cultured in Dulbecco’s modified Eagle’s medium (DMEM, HyClone, USA) containing 10% fetal bovine serum (FBS, Gibco, USA) at 37 °C with 5% CO_2_. OGD treatment was performed as previously reported [40]. Cultured astrocytes were rinsed twice and incubated in serum- and glucose-free DMEM medium (Gibco) in a hypoxia chamber (Thermo Fisher Scientific, USA) filled with premixed gas (95% N_2_ and 5% CO_2_) and maintained at 37 °C.

### Small interfering RNA transfection

After the astrocytes reached 30–50% confluence, they were transfected with siRNAs specific for GPX1 or NC as controls (GenePharma, China) using Lipofectamine 2000 (Invitrogen, USA) according to the manufacturer’s instructions. After 48 h of transfection, the astrocytes were harvested for further experiments. The sequences of the siRNAs targeting GPX1 were: 5′-GUU UCC UCU AAA CCU ACG ATT-3′ and 5′-UCG UAG GUU UAG AGG AAA CTT-3′. The sequences of the negative control were: 5′-UUC UCC GAA CGU GUC ACG UTT-3′ and 5′-ACG UGA CAC GUU CGG AGA ATT-3′.

### Flow cytometric analysis of apoptosis

Cell apoptosis was assessed using an annexinV-fluorescein isothiocyanate (FITC) apoptosis detection kit (Roche Diagnostics, Germany) according to the manufacturer’s instructions. Briefly, after the treatment, astrocytes were washed with PBS and collected into new tubes. After gently resuspending in annexinV binding buffer, the cells were incubated with annexinV-FITC/PI in the dark for 15 min and detected by flow cytometry using a BD FACSCantoll (Becton, Dickinson and Company, USA).

### Western blotting

Proteins (25 μg) were separated by 10% sodium dodecyl sulfate-polyacrylamide gel electrophoresis (SDS-PAGE) and then transferred to nitrocellulose membranes (Hybond, Amersham). The membranes were blocked with 5% nonfat milk for 1 h at room temperature and then incubated with specific antibodies against GPX1 (1:2000, Proteintech, USA), GADPH (1:20000, Cell Signaling Technologies, USA), phospho-Src family (Tyr416), phospho-PI3 kinase p85 (Tyr458)/p55 (Tyr199), phospho-Akt (Ser473), and phospho-p44/42 MAPK (Erk1/2) (Thr202/Tyr204) (1:2000, all from Cell Signaling Technologies, USA) overnight at 4 °C. After more than three washes, the membranes were incubated with the corresponding horseradish peroxidase (HRP)-conjugated secondary antibodies. Relative protein expressions were detected by chemiluminescence using the ECL kit (Thermo Fisher Scientific, USA). Quantity One software (Bio-Rad, USA) was used to quantify the relative protein expression.

### Immunofluorescence

Astrocytes were seeded into confocal culture dishes to explore the effect of allicin on GPX1 expression. They were pretreated with 50 μM allicin and then exposed to OGD or normal conditions for 4 h. After washing in PBS, astrocytes were fixed in 4% paraformaldehyde (PFA) for 15–20 min. Then, the astrocytes were permeabilized with 0.1% Triton™ X-100 for 5 min. The cells were then blocked with 1% bovine serum albumin (BSA) for 1 h and reacted with rabbit monoclonal antibody against GPX1 (1:200, Proteintech, USA) overnight at 4 °C. Then, the astrocytes were stained with goat anti-rabbit secondary antibody (1:100, Invitrogen, USA) and DAPI (1:1000, Sigma-Aldrich, USA). Finally, the excess dye was washed off with PBS, and the astrocytes were observed by a fluorescence microscope (DMi8 MAC, Leica).

### Measurement of intracellular ROS levels

To explore the effect of allicin on intracellular ROS levels, astrocytes pretreated with 50 μM allicin were exposed to OGD as previously described or normal conditions for 4 h. Intracellular ROS levels were analyzed using a Reactive Oxygen Species Assay Kit (Beyotime Biotechnology, China), according to the manufacturer’s instructions. Following treatment, the cells were observed using a fluorescence microscope (Leica DMi8 MAC, Germany).

### Lipid peroxidation assay

Malondialdehyde (MDA) levels were measured according to the manufacturer’s protocol (Beyotime, China). To remove debris, astrocytes were harvested by trypsinization, and the lysed astrocytes were centrifuged at 12,000*g* for 10 min. MDA levels and protein content were measured in the supernatant. The protein concentration was quantified using a BCA assay kit (Beyotime, China), and MDA levels were then normalized to those in the control group.

### Adeno-associated virus infection

A short hairpin RNA (shRNA) target against GPX1 (LV-GPX1-shRNA) or a control hairpin (LV-Con-shRNA) was constructed and synthesized for the local depletion of GPX1 in vivo. LV-GPX1 was used to increase the exogenous expression of GPX1 in vivo, and LV-Con was used as the control vector. All viral vectors contained the cDNA sequence for enhanced green fluorescent protein. Three days before modeling, LV was injected into the lateral ventricle of the mice, and subsequent experiments were conducted.

### Electrophysiology

An EPC-10 amplifier (HEKA Electronic, Germany) was used to record the electrophysiological signals. The resistance of the patch pipette was 4–5 MΩ, and the intracellular solution contained (mmol/L): 110 choline chloride, 2.5 KCl, 20 glucose, 1.3 NaH_2_PO_4_, 0.5 CaCl_2_, 7 MgCl_2_, 25 NaHCO_3_, and 1.3 Na–ascorbate. To elicit the total cell currents, −80 to +80 mV was applied more than 1 s from the retention potential of 0 mV, according to the voltage ramp protocols. Cell adhesion recording was then assessed in the high K+ solution to move the resting membrane toward 0 mV. A negative pressure of −45 mmHg was also applied to stretch the plasma membrane by a recording electrode that was applied syringe to be connected to a digital manometer.

### Statistical analysis

All data were statistically analyzed using GraphPad Prism 8.3.0 software. Each experiment was performed at least in triplicate for each condition, and all data are presented as the mean ± SD. The Student’s *t*-test (two-tailed) was used to compare data between the two groups. One-way analysis of variance (ANOVA) followed by the Holm–Sidak post hoc test and two-way ANOVA followed by Bonferroni’s post hoc multiple comparison tests were used for the comparison of three or more groups. A *p*-value of <0.05 was considered statistically significant. The proteomics data were deposited in the iProX database (https://www.iprox.cn/, protein ID: IPX0003704000).

### Supplementary information

Supplemental materials

Western Blots

## Data Availability

The proteomics data were deposited in the iProX database (https://www.iprox.cn/, protein ID: IPX0003704000).
