# Supplementary material for: Allicin promotes functional recovery in ischemic stroke via glutathione peroxidase-1 activation of Src-Akt-Erk
Source: Cell Death Discov. 2023 Sep 6;9:335. doi: 10.1038/s41420-023-01633-5 (PMC10482956; doi:10.1038/s41420-023-01633-5)
Supplement: Supplementary file 1 — Supplemental materials [file 41420_2023_1633_MOESM1_ESM.docx]

**Allicin Promotes Functional Recovery in Ischemic Stroke via Glutathione Peroxidase-1 Activation of Src-Akt-Erk**

Fei Zhuang^1^**^†^**, Xin Shi^1^**^†^**, Sen Qiao^2^**^†^**, Bin Liu^3^, Zhimei Wang^4^, Huanhuan Huo^1^, Feng liang^1^, Linghong Shen^1^, Lijuan Zhu^4^, Ben He^1^* and Hongmei Wang^4^*

1. Shanghai Chest Hospital, School of Medicine, Shanghai Jiao Tong University, Shanghai, 200030, China
2. Northwest Women's and Children's Hospital, Xi'an, 710003, China
3. Graduate school, Bengbu Medical College, Anhui, 233000, China.
4. School of Medicine, Southeast University, Nanjing, 210009, China

* Correspondence: heben@shchest.org; wanghongmei@seu.edu.cn

† These authors contributed equally to this work.

**Supplemental data**

**
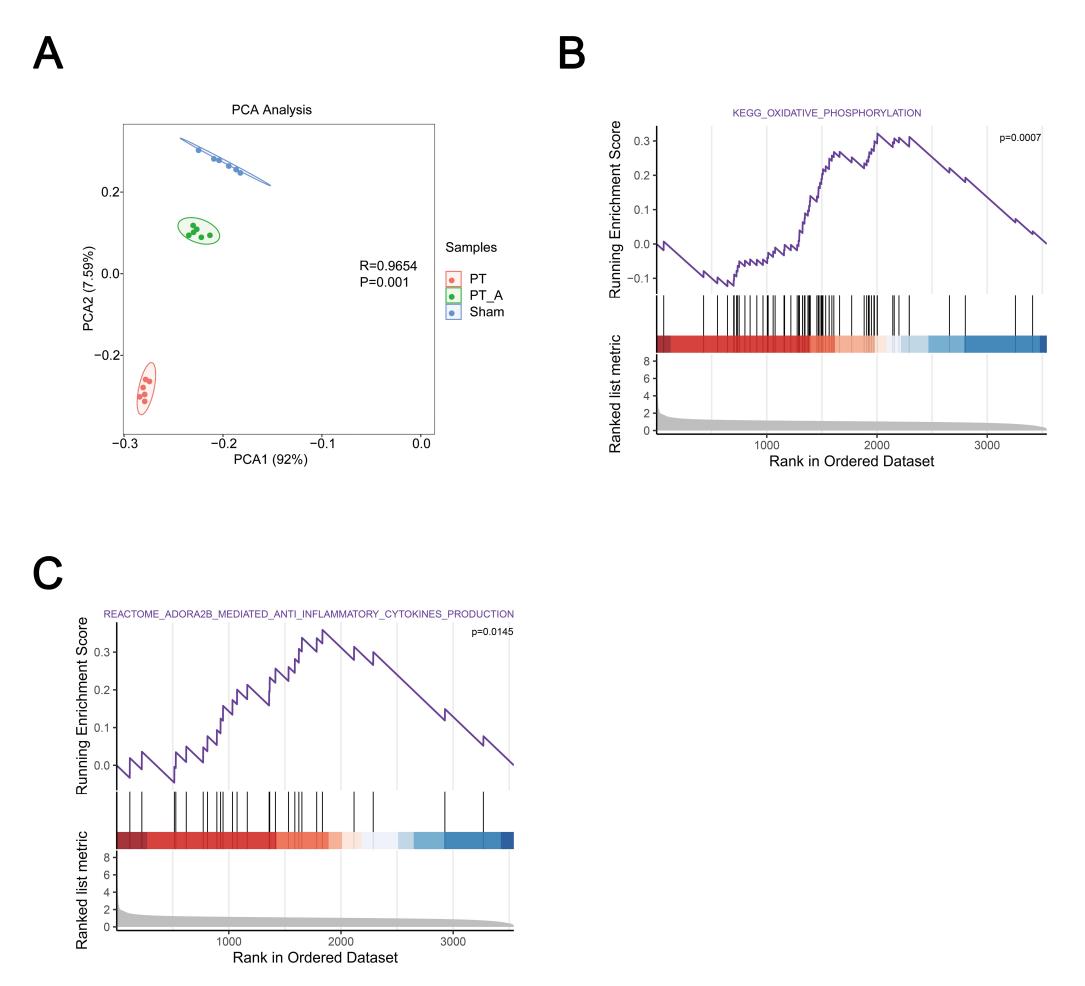
**

**Figure S1. The proteomics analysis of mice brain in ischemic stroke. (A) PCA analysis was given for proteomics. (B-C) GSEA analysis of the differentially expressed proteins between PT and PT-Allicin group.**

**
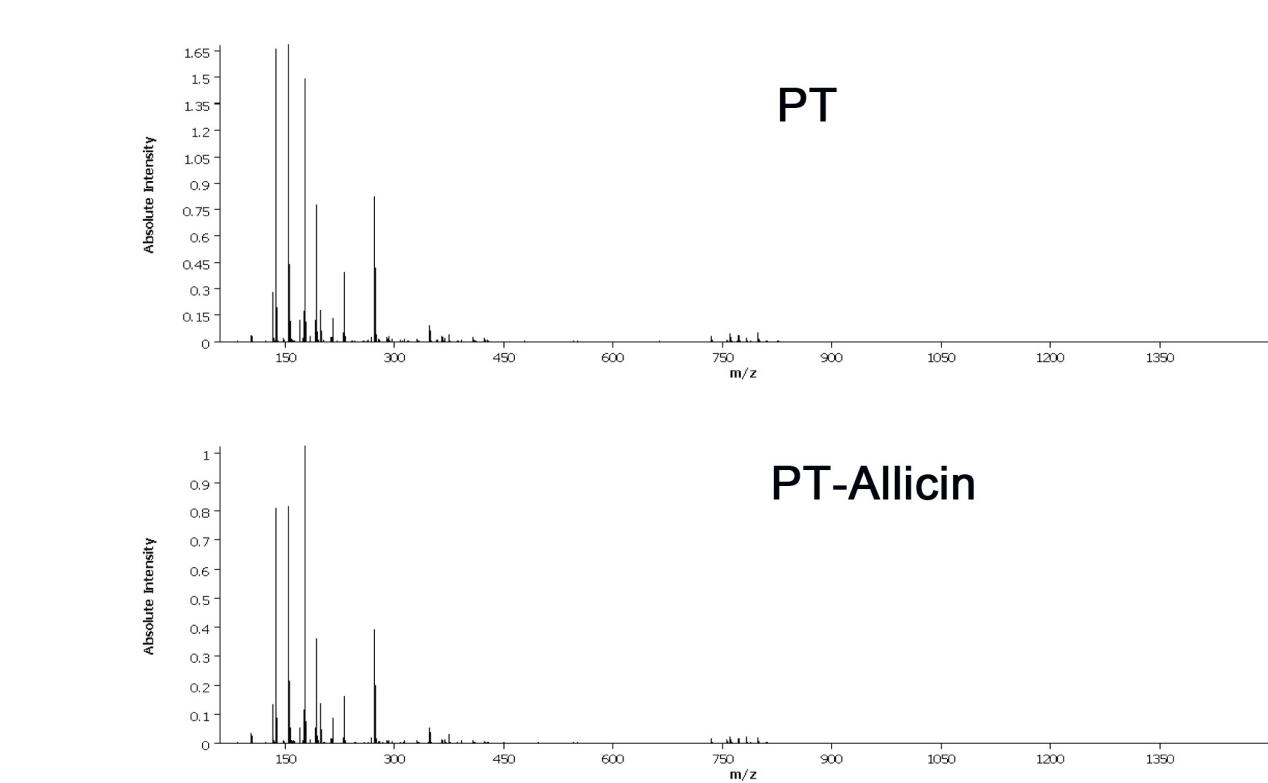
**

**Figure S2. MALDI mass spectrum of the brain in PT and PT-Allicin group with DHB matrix.**

**
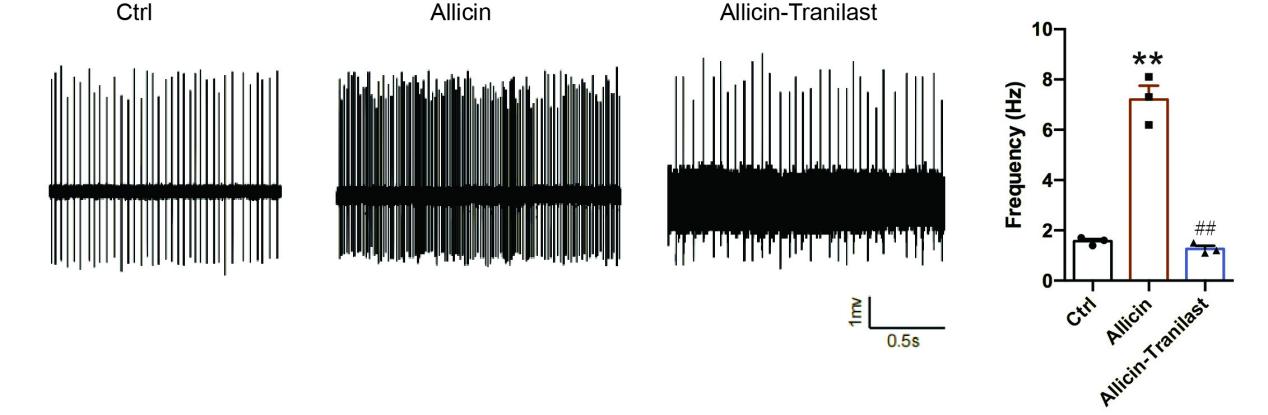
**

**Figure S3. Allicin activated the ion channel TRPV2 in astrocytes. Allicin increased the frequency of discharge; meanwhile, tranilast (75 μm) inhibited the frequency of discharge, which was induced by allicin traces (left) and bar plots (right) showing firing rates of astrocytes (for the artificial cerebrospinal fluid (ACSF) group (Ctrl). **P < 0.01 compared to Ctrl, and ##P < 0.01 compared to Allicin.**


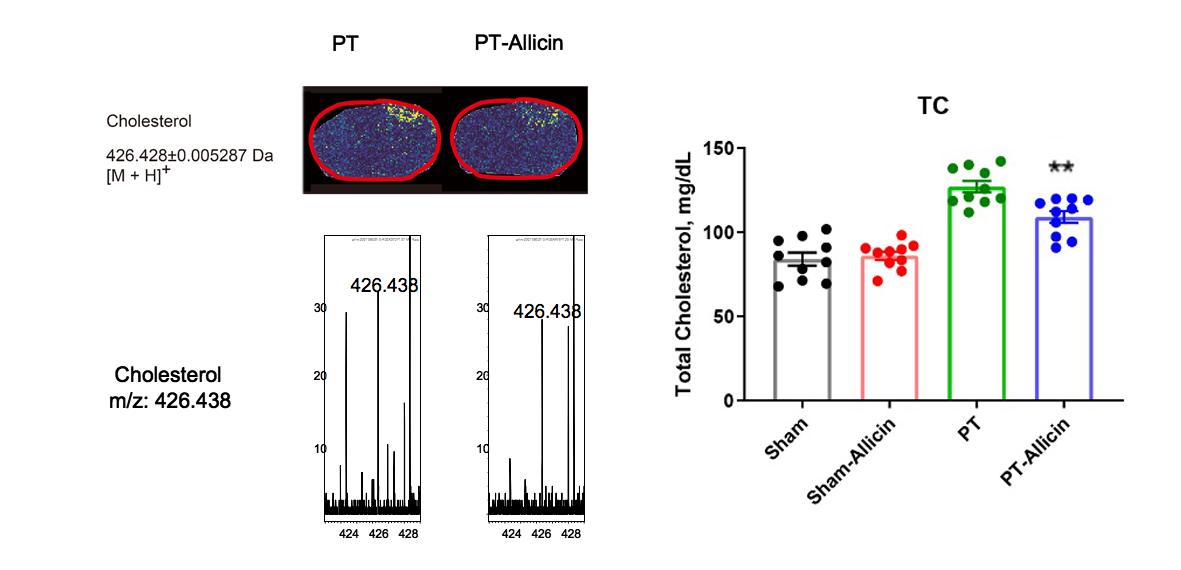


**Figure S4. Changes of total cholesterol (TC) level in allicin-treated peri-infarct cortex.** TC level in PT and PT+Allicin groups were detected by MALDI mass spectrum and ELISA kit respectively.

**Tabel S1** 308 proteins showed significant differences between PT and PT-Allicin groups (P-value < 0.05 and fold change > 2).

**Tabel S2** 126 differentially expressed metabolites between PT and PT-Allicin groups (P-value < 0.05 and fold change > 2).

**Tabel S3** 72 differentially expressed proteins in astrocytes between OGD and OGD-Allicin groups (P-value < 0.05 and fold change > 2).
