# Supplementary material for: Allicin promotes functional recovery in ischemic stroke via glutathione peroxidase-1 activation of Src-Akt-Erk
Source: Cell Death Discov. 2023 Sep 6;9:335. doi: 10.1038/s41420-023-01633-5 (PMC10482956; doi:10.1038/s41420-023-01633-5)

Allicin Promotes Functional Recovery in Ischemic Stroke via Glutathione Peroxidase-1 Activation of Src-Akt-Erk

Fei Zhuang[1†], Xin Shi[1†], Sen Qiao[2†], Bin Liu[3], Zhimei Wang[4], Huanhuan Huo[1], Feng liang[1], Linghong Shen[1], Lijuan Zhu[4], Ben He[1]* and Hongmei Wang[4]*

1. Shanghai Chest Hospital, School of Medicine, Shanghai Jiao Tong University, Shanghai, 200030, China

2. Northwest Women's and Children's Hospital, Xi'an, 710003, China

3. Graduate school, Bengbu Medical College, Anhui, 233000, China.

4. School of Medicine, Southeast University, Nanjing, 210009, China

* Correspondence: heben@shchest.org; wanghongmei@seu.edu.cn

† These authors contributed equally to this work.

Supplemental Materials-original data files-original western blots

Fig. 4A Western blot showed GPX1-shRNA obviously decreased GPX1 expression in the cerebral cortex of mice. n=6.

Fig 4A

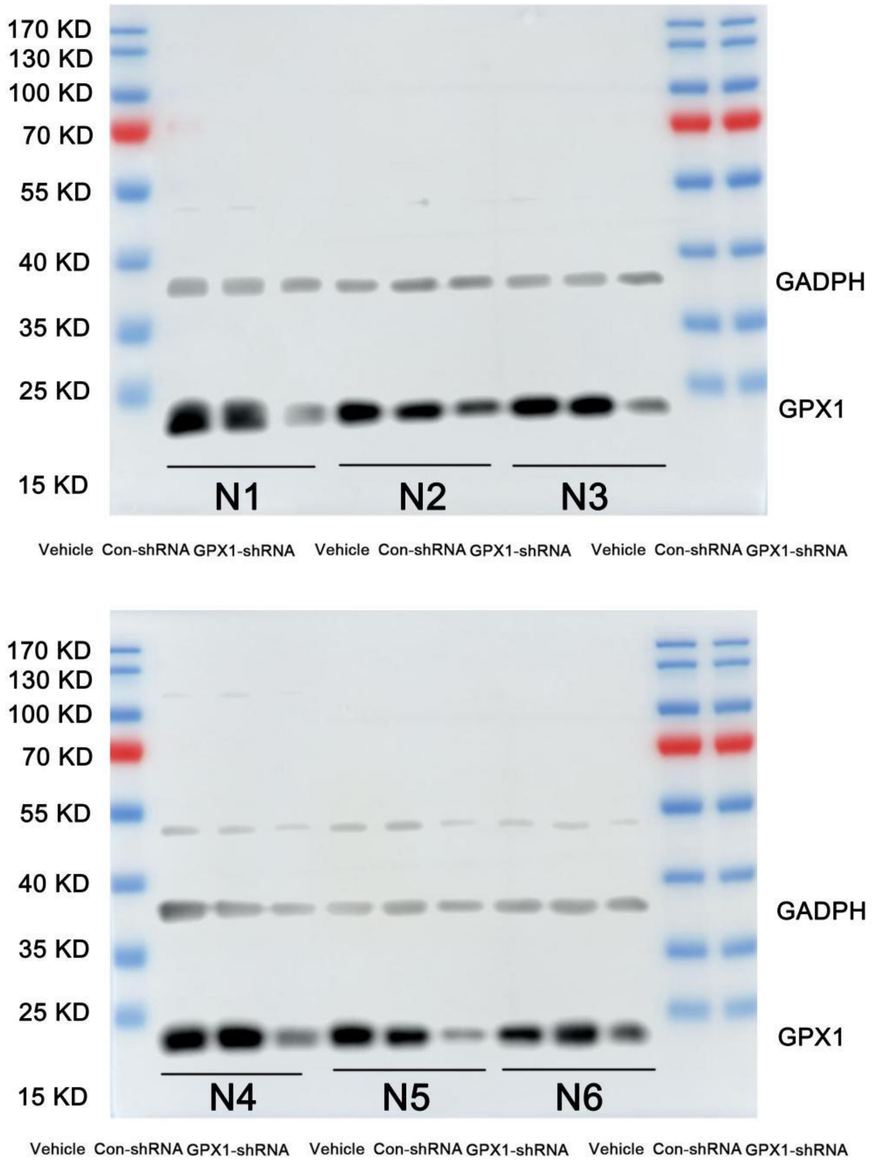

Fig. 5B Western blots further confirmed that allicin effectively reversed the inhibitory effect of hypoxia on GPX1 expression. n=6.

Fig 5B

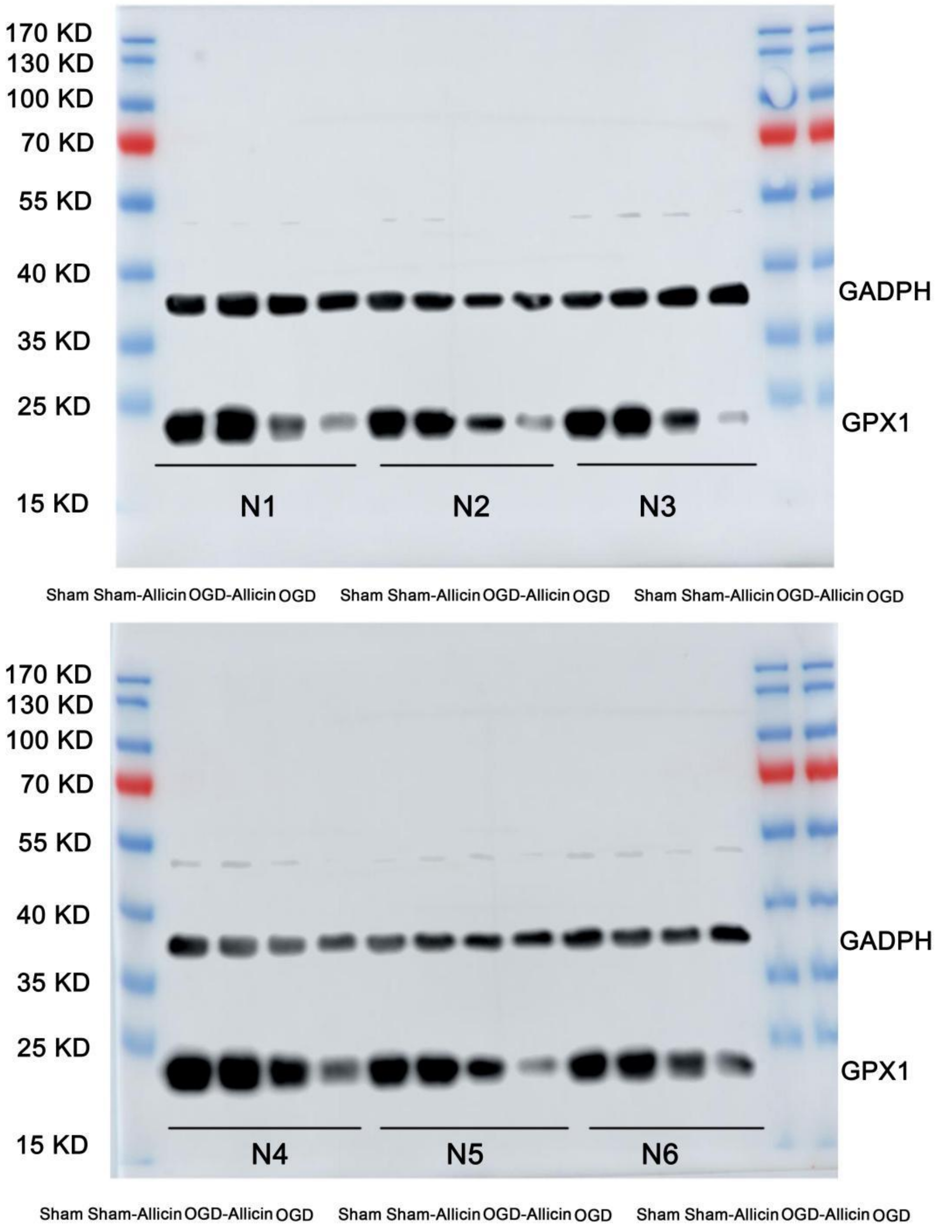

Fig. 5F Western blotting analysis showed that allicin promoted GPX1 expression in OGD-Allicin group, and obviously increased Src-Akt-Erk phosphorylation compared with OGD-NC group.

Fig 5F

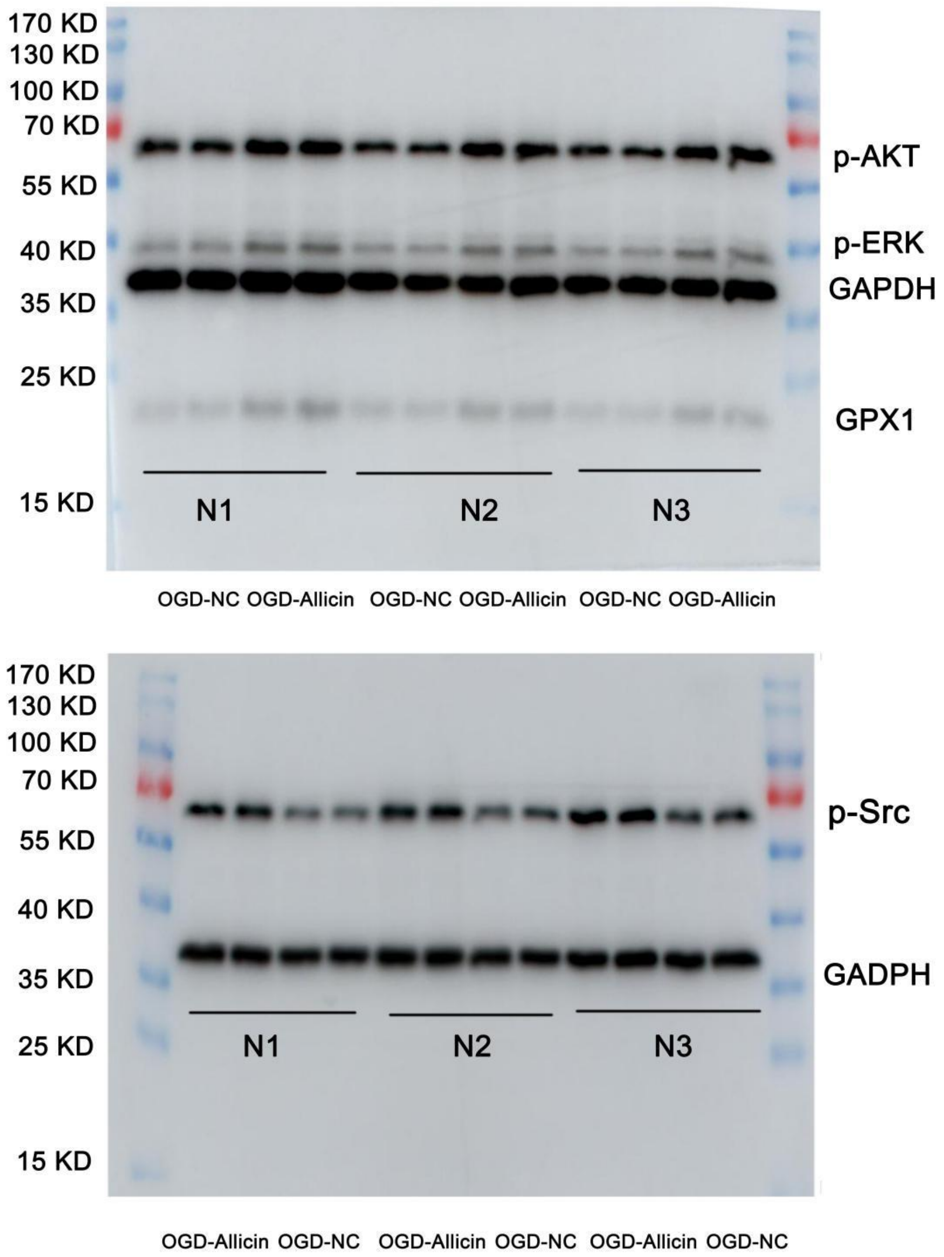

Fig 5F-2

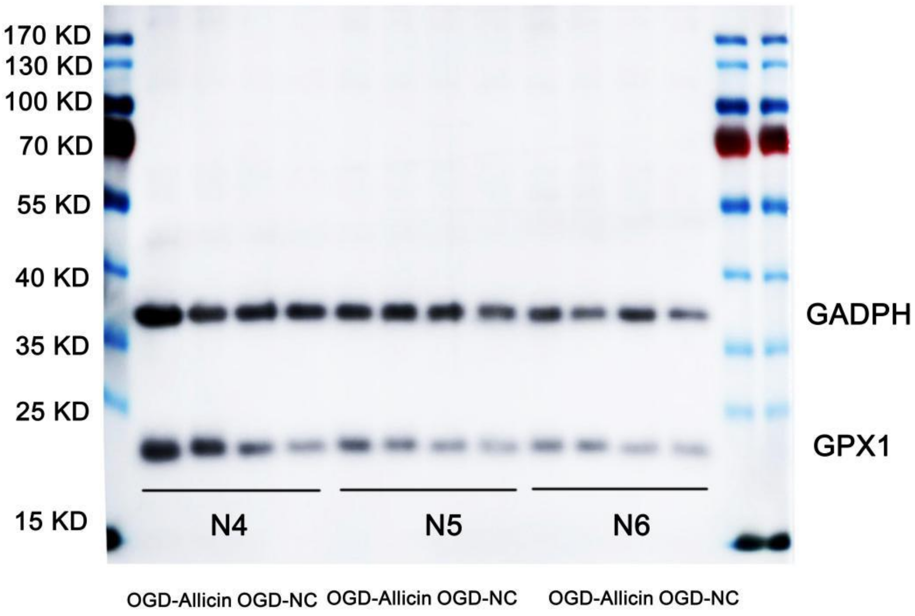
170 KD
130 KD
100 KD
70 KD
55 KD
40 KD
35 KD
25 KD
15 KD
GADPH
GPX1
N4
N5
N6
OGD-Allicin OGD-NC OGD-Allicin OGD-NC OGD-Allicin OGD-NC

Fig. 5G Western blotting analysis revealed that siRNA-GPX1 obviously decreased Src-Akt-Erk phosphorylation compared with NC group. n=6.

Fig 5G

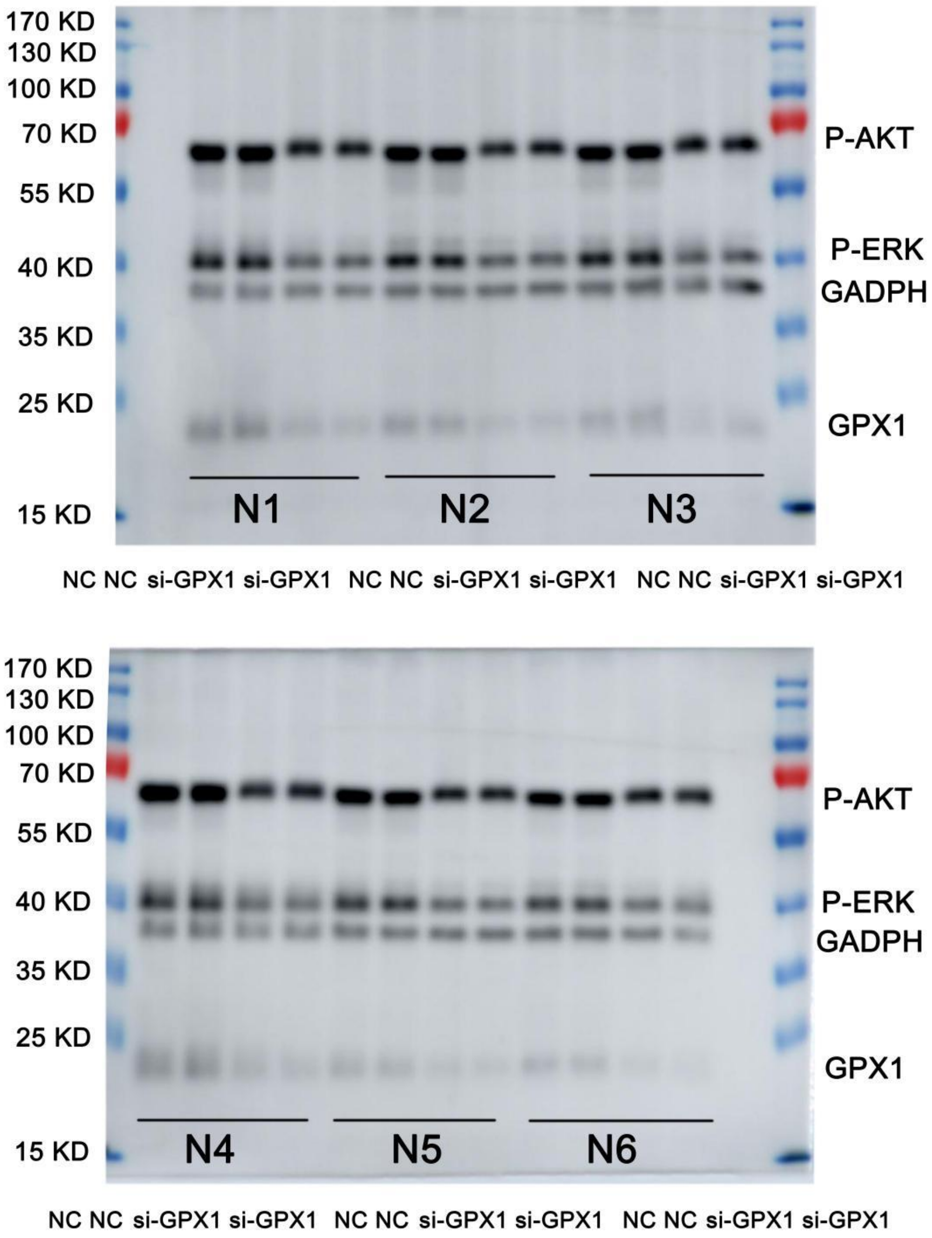

Fig 5G

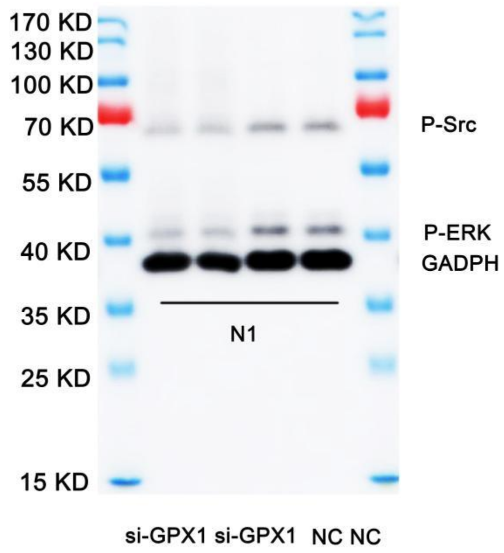

170 KD
130 KD
100 KD
70 KD
55 KD
40 KD
35 KD
25 KD
15 KD
P-Src
P-ERK
GADPH
N1
si-GPX1 si-GPX1 NC NC

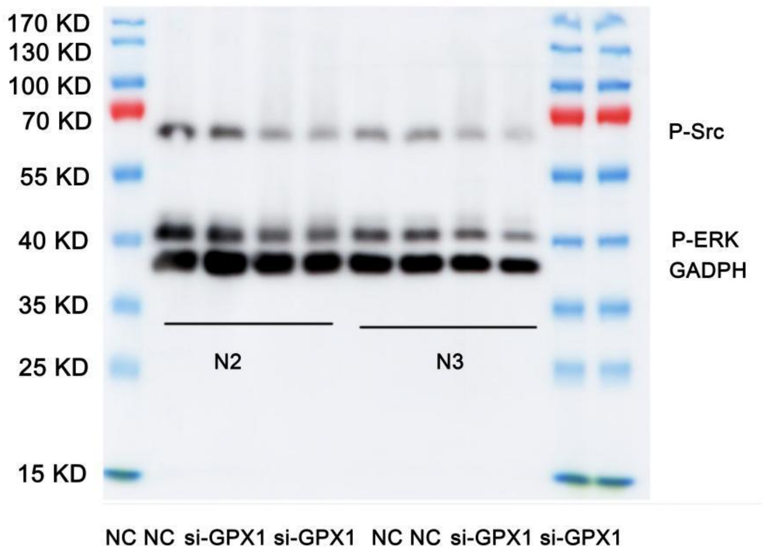

170 KD
130 KD
100 KD
70 KD
55 KD
40 KD
35 KD
25 KD
15 KD
P-Src
P-ERK
GADPH
N2
N3
NC NC si-GPX1 si-GPX1 NC NC si-GPX1 si-GPX1

Fig. 7B Western blot showed the LV-GPX1 significantly increased the expression level of GPX1 in the cerebral cortex of mice. n=6.

Fig 7B

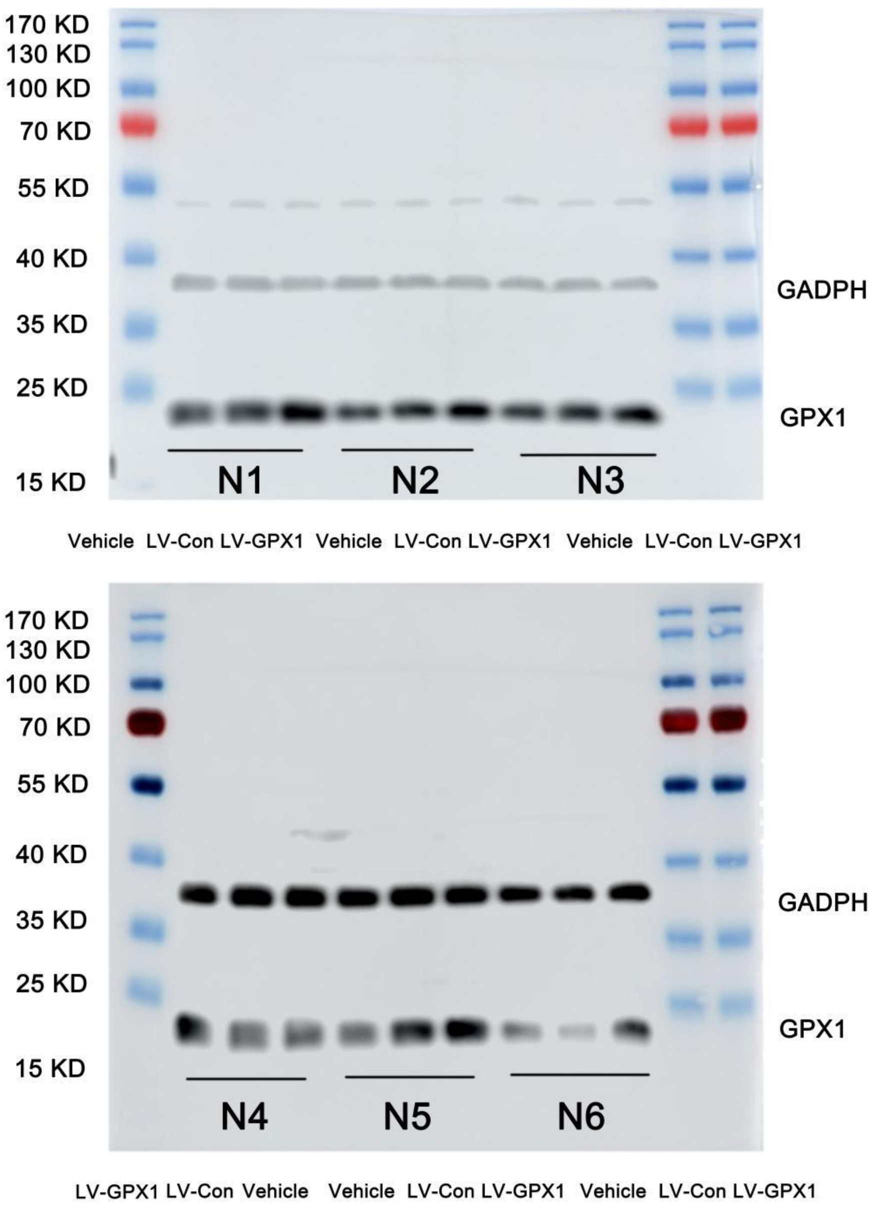

Supplement: Supplementary file 2 — Western Blots [file 41420_2023_1633_MOESM2_ESM.pdf]
